# Immune responses in mildly versus critically ill COVID-19 patients

**DOI:** 10.3389/fimmu.2023.1077236

**Published:** 2023-01-30

**Authors:** Hamid Nasrollahi, Atefe Ghamar Talepoor, Zahra Saleh, Mahsa Eshkevar Vakili, Paria Heydarinezhad, Narges Karami, Maryam Noroozi, Seppo Meri, Kurosh Kalantar

**Affiliations:** ^1^ Radio-Oncology Department, School of Medicine, Shiraz University of Medical Sciences, Shiraz, Iran; ^2^ Department of Immunology, School of Medicine, Shiraz University of Medical Sciences, Shiraz, Iran; ^3^ Department of Bacteriology and Immunology, University of Helsinki and Diagnostic Center of the Helsinki University Hospital, Helsinki, Finland; ^4^ Autoimmune Diseases Research Center, Shiraz University of Medical Sciences, Shiraz, Iran

**Keywords:** SARS-CoV-2, COVID-19, immune response, cytokine storm, T cells, antibodies, inflammation, therapeutic targets

## Abstract

The current coronavirus pandemic (COVID-19), caused by SARS-CoV-2, has had devastating effects on the global health and economic system. The cellular and molecular mediators of both the innate and adaptive immune systems are critical in controlling SARS-CoV-2 infections. However, dysregulated inflammatory responses and imbalanced adaptive immunity may contribute to tissue destruction and pathogenesis of the disease. Important mechanisms in severe forms of COVID-19 include overproduction of inflammatory cytokines, impairment of type I IFN response, overactivation of neutrophils and macrophages, decreased frequencies of DC cells, NK cells and ILCs, complement activation, lymphopenia, Th1 and Treg hypoactivation, Th2 and Th17 hyperactivation, as well as decreased clonal diversity and dysregulated B lymphocyte function. Given the relationship between disease severity and an imbalanced immune system, scientists have been led to manipulate the immune system as a therapeutic approach. For example, anti-cytokine, cell, and IVIG therapies have received attention in the treatment of severe COVID-19. In this review, the role of immunity in the development and progression of COVID-19 is discussed, focusing on molecular and cellular aspects of the immune system in mild vs. severe forms of the disease. Moreover, some immune- based therapeutic approaches to COVID-19 are being investigated. Understanding key processes involved in the disease progression is critical in developing therapeutic agents and optimizing related strategies.

## 1 Background

The coronavirus disease 2019 (COVID-19), caused by the severe acute respiratory syndrome- coronavirus- 2 (SARS-CoV-2), is a viral infectious disease spread worldwide (1). The outbreak of COVID-19 has led to extensive global morbidity and mortality (2). The Coronaviridae family contains α and β coronaviruses capable of causing both mild and severe respiratory diseases in wild animals and humans. SARS-CoV, MERS-CoV and SARS-CoV-2 are positive- sense single-stranded RNA β-coronaviruses causing severe respiratory tract infections in humans (3, 4).

SARS-CoV-2 infection can have a variable clinical picture ranging from asymptomatic to severe. In about 5% of patients, there is a risk of death, while the overall mortality is about 1% (5). With increasing vaccine coverage the numbers are getting smaller. SARS-CoV-2 can enter and infect epithelial, and possibly endothelial cells expressing the receptor angiotensin- converting enzyme 2 receptor (ACE2) and, in certain cells, neuropilin-1 (6, 7).

During the initial exposure, the virus infects epithelial cells in the upper respiratory tract through the ACE2 receptor. At this stage, the innate and adaptive immunity try to inhibit the virus at the mucosal sites and prevent the progression of the disease by various mechanisms. These include the production of mucus and antiviral metabolites, interferons and other cytokines, as well as IgA, when there is preexisting immunity to the virus.

SARS-CoV-2 is able to inhibit innate and adaptive immune responses in various ways. It can replicate unchecked in the respiratory tract and eventually spread to the lungs (8, 9). COVID-19 patients may develop a systemic disease with extrapulmonary manifestations. Systemic manifestations during a later stage of COVID-19 are linked to lymphopenia, increased amounts of neutrophils, cytokine release syndrome (CRS) or cytokine storm, activation of complement and coagulation cascades and elevated levels of inflammatory chemokines and alarmins (10, 11). Several studies have shown that male gender, higher age, obesity, diabetes, hematological cancer, autoimmune diseases (e.g., psoriasis), hypertension (especially pulmonary arterial hypertension), and cardiovascular, cerebrovascular and chronic kidney diseases are risk factors closely related to the severity and outcomes of COVID-19 infection (12–16).

Although innate and adaptive immunities are linked, they both also have different cell types with varying functions (17). The innate immune system includes monocytes, macrophages, dendritic cells (DC), myeloid-derived suppressor cells (MDSC) and innate lymphoid cells (ILCs). ILCs can be divided into different groups: natural killer (NK) cells, 3 subsets of helper like ILCs (ILC1, 2 and 3), fetal lymphoid tissue- inducer (LTi) cells and a population of innate intraepithelial lymphocytes (IELs) in the intestine that express T cell markers (18). In addition, the innate response includes a number of molecules, e.g. the entire complement system with more than 40 different soluble or cell-bound components. Innate immune cells expressing pattern-recognition receptors (PRRs) can detect pathogen-associated molecular patterns (PAMPs) of invading viruses to develop and maintain innate immune responses by secreting interferons (IFNs) of type I (IFN-α and IFN-β) and type III (IFN-λ), as well as pro-inflammatory cytokines and chemokines (19, 20). The adaptive immune system comprises B cells, CD4^+^ T helper and CD8^+^ cytotoxic T cells. CD4^+^ T cells orchestrate the immune response by producing cytokines and providing help to other immune cells. CD8^+^ T cells kill virally infected cells, and B cells develop and produce antigen-specific IgM, IgG and IgA antibodies (21). Larger amounts of specific antibodies are produced by plasma cells that locate themselves into niches in the bone marrow.

Given that appropriate innate and adaptive immune responses are required to control and contain COVID-19 infection and prevent the disease in the long term, it is critical to recognize both types of responses to SARS-CoV-2. This review aims to shed light on the molecular and cellular players of the immune system in COVID-19 pathogenesis. Accordingly, the molecular and cellular compartments involved in the innate and adaptive immune responses to SARS-CoV-2 are thoroughly reviewed (A brief comparison of immune responses in mildly versus critically ill COVID-19 patients has been provided in **Table 2**). Special emphasis is on delineating differences that distinguish severe disease forms from those of milder forms. Why do certain individuals suffer from serious lung complications and systemic effects, and what is the contribution of the immune system itself in a vicious circle of inflammation in response to a viral infection?.

## 2 The relationship between the innate immune system and the progression of COVID-19

### 2.1 Neutrophils

**Figure 1:**
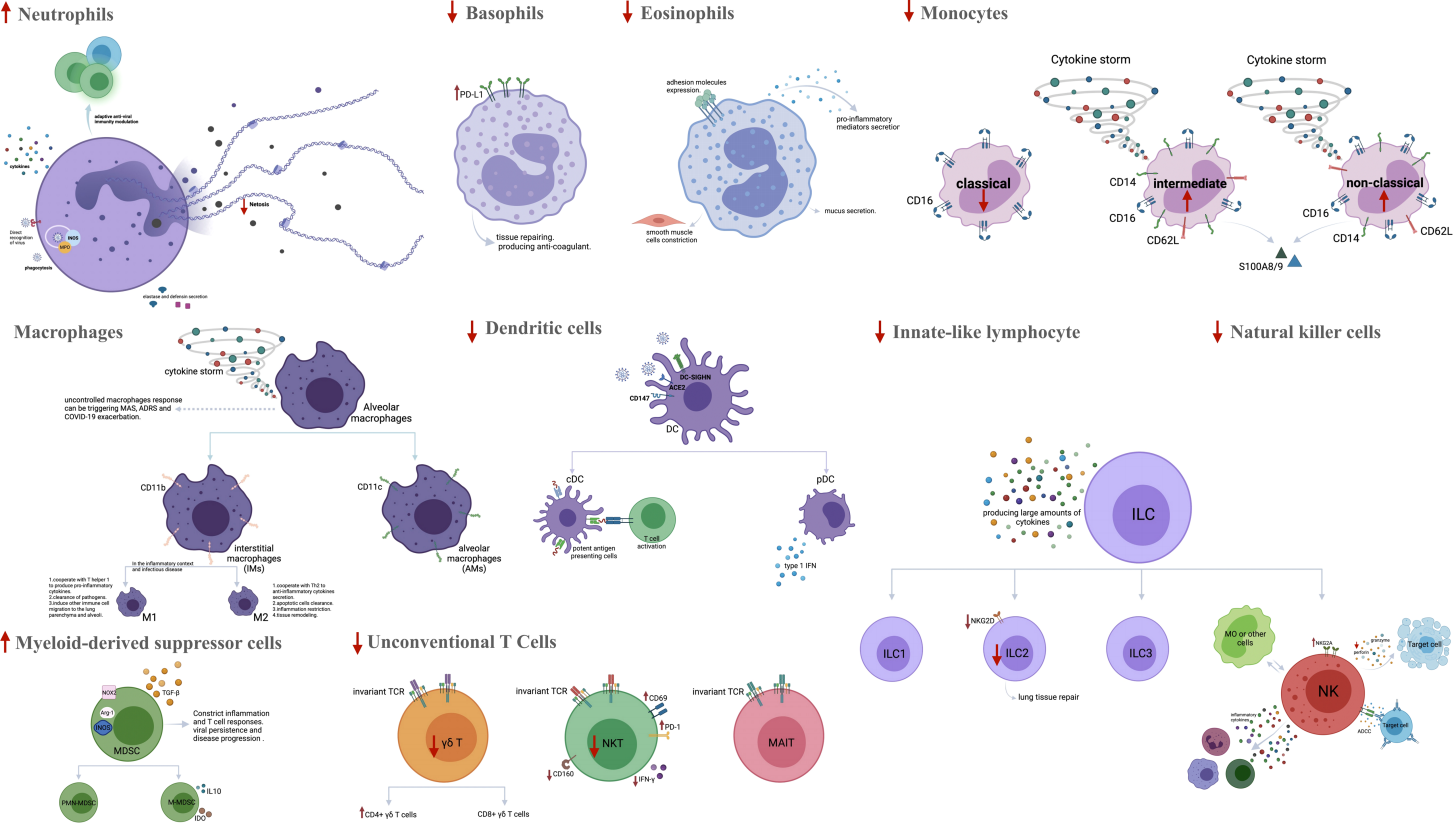
The frequency and function of innate immune cells in severe COVID-19. SARS-CoV-2 infects cells that present the surface receptor ACE2. It is recognized by tissue-resident immune cells in the lungs, which provoke a local immune response. More innate immune cells are recruited from the blood by chemokines and complement activation products. The amounts of neutrophils, CD16^+^ monocytes, M1 macrophages and MDSCs are bigger in severe COVID-19 patients, while the percentages of basophils, eosinophils, M2 macrophages, classical monocytes, DCs, ILCs, NK cells and unconventional T cells are reduced. Activated neutrophils can amplify inflammation by secretion of pro-inflammatory cytokines, MPO, elastase and defensins. They can also promote NET formation and Th17 cell differentiation. Activated basophils and eosinophils appear to predispose the development of ARDS by releasing inflammatory and vasoactive substances. Peripheral CD16^+^ monocytes and M1 macrophages are involved in a hyper-inflammatory response and cytokine storm during the inflammatory phase in severe COVID-19. Significant decrease in viral sensing and IFN signaling as well as suppressed antigen presentation by both pDC and cDC have been reported in severe COVID-19. Aberrant or ineffective responses of ILCs (ILC1, ILC2, ILC3 and NK cells), MDSCs and unconventional T cells (MAIT cells, γδ T cells) also have role in promoting inflammation in severe COVID-19. ACE2, Angiotensin-converting enzyme 2; MDSCs, Myeloid-derived suppressor cells; DC, Dendritic cell; ILCs, Innate-like lymphocyte; NK cell, Natural killer cell; MPO, Myeloperoxidase; NET, Neutrophil extracellular trap; Th, T helper; ARDS, Acute respiratory distress syndrome; MAS, Macrophage activation syndrome; PD-L1, Programmed cell death ligand 1; IFN: Interferon; cDC, Classical DC; pDC, Plasmacytoid DCiNOS, Inducible nitric oxide synthase; PMN-MDSCs, Polymorphonuclear-MDSCs; M-MDSCs, Monocytic-MDSCs; ADCC, antibody-dependent cellular cytotoxicity; NKG2A, NK cell lectin-like receptor subfamily C member 1. Figure was created by BioRender (Toronto, ON, Canada).

SARS-CoV-2 infection is marked by neutrophil migration into pulmonary capillaries and alveolar spaces, leading to acute capillaritis, fibrin deposition and neutrophilic mucositis (22). Neutrophils display antiviral activity through different mechanisms, including direct, antibody or complement-mediated recognition of the virus and phagocytosis and secretion of cytokines, myeloperoxidase (MPO), elastase and defensins. They can also form neutrophil extracellular traps (NET) and modulate adaptive antiviral immunity (23–25). Recent studies have found increased neutrophil counts in the peripheral blood of non-surviving and severely ill COVID-19 patients compared to mildly ill cases. In addition, increased numbers of low-density neutrophils have been found in the blood of acute COVID-19 patients (26, 27). Different populations of granulocytes in the blood could reflect emergency myelopoiesis. Some of the granulocytes (N2-type) could be immunosuppressive and cause impaired lymphocyte responses during acute disease (28). Previous studies have also observed a high neutrophil- to- lymphocyte ratio (NLR) in critically ill COVID-19 patients compared to cases with mild disease. Therefore, NLR has been introduced as an independent risk factor for severe disease (27, 29, 30). High levels of MPO and citrullinated histone H3 (Cit-H3), serum markers of NETs and indicators of neutrophil activation have also been described in severe COVID-19 (31–33). It has thus been concluded that neutrophilia and excessive formation of NETs, as well as the release of reactive oxygen species (ROS), in severe COVID-19 trigger cytokine release and respiratory failure, contributing to disease severity (22, 34, 35). The changes in innate immune cells in COVID-19 are summarized in **Figure 1**.

### 2.2 Monocytes

Monocytes are a pivotal component of the innate immune response. They participate in inflammatory responses, phagocytosis, antigen presentation and other immune functions (36, 37). Human monocytes are classified into classical (CD14^high^ CD16^−^), intermediate (CD14^high^CD16^+^) and non-classical (CD14^low^ CD16^+^) subsets (38). Intermediate and non-classical monocytes are known as the “inflammatory” subsets due to their potent pro-inflammatory activity. They contribute to various conditions such as asthma, coronary artery disease, Crohn’s disease, sepsis and hepatitis B (39). Recent studies have demonstrated that inflammatory monocytes are involved in the hyper-inflammatory response and cytokine storm observed during the infectious and inflammatory phases of severe COVID-19 (40). The results indicate that the absolute numbers of all blood and CD16^-^ classical monocytes decrease in severely ill COVID-19 patients, while the levels of the inflammatory subsets increase in patients in comparison to the healthy controls (41). One study showed an increased percentage of inflammatory monocytes expressing S100A8+ S100A9 (calprotectin), CD14 and CD62L, possibly correlating with a profibrotic differentiation pattern in severe COVID-19 as compared to mild disease (42). Furthermore, a significant increase in the frequency of CD14^high^ CD16^+^ monocytes producing IL-6 was reported in the peripheral blood of COVID-19 patients in the ICU compared to those without ICU hospitalization (43, 44). An increased number of inflammatory monocytes in patients with mild disease has also been reported in several studies. This could be considered as an indicator of viral control in such patients (45, 46).

### 2.3 Macrophages

The function of alveolar macrophages in the lungs is to preserve homeostasis by removing dead cells and invading pathogens (47). Under steady-state, two distinct populations of macrophages, the interstitial macrophages (IMs) and alveolar macrophages (AMs), are present in the lungs (48, 49). Interstitial macrophages are characterized as CD11b^+^CD11c^-^ cells localized in the parenchyma, while AMs are identified as CD11b^-^CD11c^+^ macrophages residing in the air space lumen (50). In inflammation and infectious diseases, alveolar macrophages are polarized into the M1 or M2 subgroup (47). M1 macrophages cooperate with T helper (Th) 1 cell to produce pro-inflammatory cytokines and clear pathogens. They also induce the migration of other immune cells to the lung parenchyma and alveoli. M2 macrophages are associated with Th2 cells to increase the secretion of anti-inflammatory cytokines and clearance of apoptotic or senescent cells. Thus, the M2 cells restrict inflammation and promote tissue remodeling (47, 51).

Based on recent data, macrophages are considered promoters of the cytokine storm during infection by SARS-CoV-2. They are associated with severe COVID-19 with a high risk of death (52, 53). Moreover, the cytokine storm, mainly macrophage activation syndrome (MAS), in addition to complement and neutrophil activation, contributes to COVID-19-associated adult respiratory distress syndrome (ARDS) and its exacerbation (54, 55). Another study reported a significant correlation between SARS-CoV-2 infection of macrophages in the spleen and lymph nodes and severe lymphocyte apoptosis in COVID-19 patients (27). Recent studies by single-cell RNA sequencing of samples recovered from bronchoalveolar lavage fluid of mildly and severely ill COVID-19 patients have revealed that SARS-CoV-2 infection leads to the recruitment of an inflammatory macrophage subset with down-regulation of type I IFN genes, which is related to disease severity (56–58). In conclusion, macrophages sense SARS-CoV-2 and respond to its threat by secreting pro-inflammatory cytokines and chemokines. However, an uncontrolled macrophage response could trigger MAS, ARDS and an exacerbation of COVID-19 (42).

### 2.4 Dendritic cells

Among other places, DCs are distributed in the respiratory tract, where they can recognize invading microbes. SARS-CoV-2 can gain entry into DCs, particularly into interstitial lung DCs, *via* ACE2, CD147 and dendritic cell-specific intracellular adhesion molecule-grabbing non-integrin (DC-SIGN) receptors (59). Classical myeloid DCs (cDCs) and plasmacytoid DCs (pDCs) are two distinct DC subsets in humans. The former serve as potent antigen- presenting cells (APCs), while the latter is a specialized subset that can produce type 1 IFN (60, 61). It has been found that pDC along with viral sensing, interferon signaling and antigen presentation are more defective in severe versus mild COVID-19 (62–68). Further impairment in DC maturation and function in severe vs. mild disease may be associated with a worse disease (69). In addition, another study reported that the frequency of DCs in the blood of COVID-19 patients decreased compared to healthy controls. It was found that DCs from individuals with acute COVID-19 were functionally impaired in maturation and in the ability to activate T cells (43, 70). Two recent studies found both types of DCs (cDCs and pDCs) to be much less abundant in the blood samples of people with severe COVID-19 than in those with mild COVID-19 (71, 72).

### 2.5 Innate-like lymphocytes

ILCs are effector cells that detect environmental stimuli and contribute to early immune responses by producing large amounts of cytokines (73). They are categorized into four main groups: NK cells, ILC1, ILC2, and ILC3 (74, 75). Transcriptionally and functionally, the ILC1, ILC2, and ILC3 resemble Th1, Th2, and Th17 cells, respectively, while NK cells resemble CD8^+^ T cells (73). However, little is known about the distribution and function of different ILC subsets in COVID-19. Recent studies have reported a reduced percentage and lower absolute counts of ILCs in the blood of COVID-19 patients (73). Accordingly, results have shown a significant decrease in the frequency of ILC2 in severe compared to moderate COVID-19 (73). Furthermore, previous studies have observed that the frequency of ILC2s in COVID-19 patients negatively correlates with the level of the coagulation factor fibrinogen-derived D-dimer and the development of severe disease (76, 77). ILC2s could thus have a protective role in the disease.

Previously, it has been shown that ILC2 plays an important role in lung tissue repair during *influenza A* infection in mice (78). Therefore, it can be hypothesized that low ILC2 levels in COVID-19 patients correlate with a more severe disease outcome. One further investigation reported a decreased frequency of all ILCs, and particularly of the ILC2 subpopulation, in severe COVID-19. Also, compared to mildly diseased patients, it was observed that the expression of NKG2D^+^, the activating C-type lectin-like molecule, on ILC2s was significantly reduced in patients requiring mechanical ventilation (79). Therefore, an increase in NKG2D^+^ ILC2s, along with elevated levels of anti-inflammatory mediators, might be related to a better prognosis in severe COVID-19 (**Figure 1**).

### 2.6 Natural killer cells

NK cells are the most important innate immune cells in fighting viral infection. This is because they can induce direct lysis of target cells by producing perforin and granzyme B, secrete inflammatory cytokines, contribute to antibody-dependent cell cytotoxicity (ADCC) and interact with other immune cells such as monocytes (80). A significant decrease in the absolute number of NK cells in the peripheral blood of patients with severe COVID-19, as well as in those admitted to ICU, has been reported recently (81–83). According to Leem et al., unconventional CD56^dim^CD16^neg^ NK cells, associated with decreased cytotoxic activity, expanded in both severe and mild forms of the disease. These changes were reversed more rapidly in mild vs. severe COVID-19 (84). Another study showed that the percentage of perforin^+^ NK cells in COVID-19 patients, who were in the ICU, was reduced as compared with non-ICU patients and healthy controls (85). Previous studies have shown an increased expression of the inhibitory receptor NKG2A on NK cells in severely ill COVID-19 patients, as compared to mildly ill patients and healthy subjects (86). NKG2A expression along with decreased IFN-γ, granzyme B, and TNF-α production is associated with NK cell exhaustion and disease progression in COVID-19 patients. Further, the percentage of NK cells expressing NKG2A is reduced in COVID-19 patients, who recovered after receiving proper therapy (27, 87). Hajeer et al. found an association between two NK cell immunoglobulin-like receptors (KIR), named KIR2DS4 and KIR3DL1, with an inhibitory and activating function, respectively, and an increased risk of severe disease (88). Another study reported a significant decrease in the inhibitory KIR receptors in COVID-19 patients compared to healthy controls and a considerable increase in activating KIR receptors in patients with a severe disease (89) (**Figure 1**).

### 2.7 Basophils

Basophils are a type of granulocyte representing less than 1% of peripheral blood leukocytes (90). As they are involved in tissue repair and production of anti-coagulant factors, their depletion results in increased pneumonitis in COVID-19 (91). In recent studies, a significant decrease has been observed in the frequency and the absolute numbers of basophils in patients with severe COVID-19 (82, 92, 93). Moreover, a stronger expression of programmed cell death ligand 1 (PD-L1), an inhibitor of T cell activation, has been found on basophils in severe vs. mild COVID-19, correlating with poor disease outcomes (89).

### 2.8 Eosinophils

Eosinophils are multifunctional granulocytes involved in various inflammatory contexts, including helminth, bacterial and viral infections, tissue injury, tumor immunity and allergic diseases. They secrete pro-inflammatory mediators, express adhesion molecules and support smooth muscle cells (94, 95). Significantly decreased eosinophil counts have been found in COVID-19 patients compared to healthy controls or patients with mild disease (82, 96). A decrease in eosinophil counts is related to fever, fatigue, shortness of breath and poor outcome in COVID-19 patients (96, 97).

### 2.9 Myeloid-derived suppressor cells

In humans, MDSCs are classified into polymorphonuclear-MDSCs (PMN-MDSCs) and monocytic-MDSCs (M-MDSCs), which control inflammation and T cell responses through a high-level expression of inducible nitric oxide synthase (iNOS), arginase-1 (Arg-1), nicotinamide adenine dinucleotide phosphate oxidase (NOX2) and transforming growth factor-beta (TGF-β) (98). In viral infections, MDSC-mediated immunosuppression causes viral persistence and disease progression (99). A recent study showed a significantly higher frequency of PMN-MDSCs in the peripheral blood of patients with severe COVID-19 and in those who required ICU compared to healthy blood donors (98). This correlated with decreased antigen-specific T cell responses and poor disease outcomes (98). Another study demonstrated an increased frequency of MDSCs in both mild and severe COVID-19. According to this study, MDSC expansion during the early phase of acute infection was protective by reducing T cell hyperactivation and inflammation but could also decrease the protective immune response (100). The percentage of M-MDSCs correlated positively with neutrophil count, C-reactive protein (CRP) and D-dimer levels, hospital stay and viral RNA load and negatively with lymphocyte count and serum albumin in COVID-19 patients admitted to ICU compared to healthy controls and non-ICU patients (101). An increase in MDSC recruitment and expansion along with impaired T cell function was observed in severe COVID-19. This was related to IL-10, arginase-1 and indoleamine 2,3-dioxygenase (IDO) production by MDSCs (102, 103).

### 2.10 Unconventional T cells

Unconventional T or innate-like T cells, such as natural killer T (NKT), mucosa-associated invariant T (MAIT) and γδ T cells, exhibit characteristics of both innate and adaptive cells. They express T cell receptors (TCRs) with limited diversity and recognize alternative microbial antigens in major histocompatibility complex (MHC)-unrestricted compartments (74). Most responses of NKT and γδ T cells seem to be stimulated against pathogenic agents. In particular, the potent cytotoxic responses of these cells in patients infected with influenza A viruses, herpes viruses or lentiviruses may be essential for suppressing viral replication and regulating immunosuppressive MDSCs (104–110). Recent studies have shown a reduced frequency of NKT cells in patients with severe COVID-19 and a small PaO2/FiO2 (P/F) ratio (82, 111). An increased expansion of CD160^+^ NKT cells has been reported in mild COVID-19, possibly supporting recovery from infection by direct cytotoxicity (112). Furthermore, decreases in the levels of circulating NKT cells and IFN-γ production are observed in severe COVID-19. However, the expression levels of CD69 and PD-1 are increased in NKT cells, and a strong PD-1 expression in NKT cells continues in patients in ICU on day 15 (113). Another study showed that while the total percentage of γδ T cells decreased in COVID-19 patients, the percentage of CD4^+^γδ T cells significantly increased (114). It was suggested that the latter subset might be associated with antigen presentation and activation of adaptive immune cells (114). Based on another investigation, increased frequencies and absolute numbers of naïve-like (CD45RA^+^CD62L^+^) γδ T cells and a decreased frequency of effector-like (CD45RA^-^CD62L^−^) γδT cells were observed both in mild and severe COVID-19. This study suggested that effector-like γδ T cells might be localized in the lungs of COVID-19 patients to contribute to the immune response against the infecting virus (115) (**Figure 1**).

### 2.11 Complement system

The complement system belongs mostly to innate immunity. It can respond to invading microbes and other foreign materials (22, 116). It may recognize SARS-CoV-2 directly or with the help of antibodies. Additionally, tissue damage caused by the virus can activate complement. Complement activation can lead to acute or chronic inflammation, thrombus formation, endothelial cell dysfunction and intravascular coagulation, thus ultimately contributing to multiple organ failure (MOF) and death (117). A recent study showed prominent complement activation in the lungs, skin and blood of severely ill COVID-19 patients compared to cases with mild disease. Some individuals treated with a complement inhibitor recovered with no adverse reactions (118). Complement activation generates the pro-inflammatory polypeptides, C3a and C5a, which can recruit and activate neutrophils and monocytes. Mannose-binding lectin (MBL) interacts with SARS-CoV-2 and could consequently activate complement C3 *via* the lectin pathway (119). Through neutrophil extracellular trap (NETs) formation, neutrophils activate the alternative complement pathway and may engage an inflammatory feedback loop. Recently, a significant correlation has been found between the activation of complement component C3 and COVID-19 severity (120, 121). Similarly, an investigation showed higher levels of C3a and C5a as well as C3-fragment deposition in the lung biopsy samples of severe COVID-19 patients (122). Furthermore, in a further study, high levels of soluble C5a and an increased expression of the C5a receptor in blood and pulmonary myeloid cells were found in severe COVID-19 with acute respiratory distress syndrome compared to mildly diseased patients and controls (123). Thus, multiple features of severe COVID-19 point to the importance of complement activation in its pathogenesis, mainly during inflated inflammatory responses and effects on vascular endothelial cells, including promotion of blood coagulation and increased capillary permeability.

## 3 The relationship between the adaptive immune system and the progression of COVID-19

### 3.1 Cell-mediated immunity

CD4^+^ and CD8^+^ T cells play important roles in resolving acute viral infections and providing subsequent protection against reinfection. DCs induce polarization and maturation of naive T cells *via* the presentation of peptides in the context of MHC along with the secretion of chemokines and cytokines. Subsequently, activated T cells migrate to the site of infection, producing antiviral cytokines, chemokines and cytotoxic molecules (124). Acute and severe SARS-CoV-2 infections are associated with lymphopenia and remarkable loss of CD4^+^ and CD8^+^ T cells, which is reversed by disease recovery (125–127). Transient lymphopenia may be associated with a direct effect of SARS-CoV-2 infection on the production and/or differentiation of lymphocytes in primary lymphoid organs and their release into the circulation (115, 128). High levels of IL-6, IL-10 or TNF-alpha directly promote thymic dysfunction and indirectly promote T cell apoptosis (127, 129–132). In early studies on COVID-19, impairment in function and increased expression of markers, which are hallmarks for activation and/or exhaustion of CD4^+^ and CD8^+^ T cells, were observed in patients. The expression levels of Ki-67, PD-1, perforin and granzyme B in CD4^+^ or CD8^+^ T cells were found to be high in patients with severe disease (133, 134). Some studies have considered the PD-1/PDL-1 axis as a severity-associated biomarker that can inform about lymphocyte depletion/exhaustion (135, 136). However, it seems that the stronger expression of T cell inhibitory molecules (PD-1 and Tim-3) in severe vs. mild COVID-19 might not only indicate T cell exhaustion but could also suggest the existence of antigen-specific T cells (77, 137, 138). Recent studies, including more than 1000 COVID-19 cases, have indicated that a reduced frequency of CD4^+^ T cells is related to disease severity (139, 140). The lower proportion of SARS-CoV-2-specific CD4^+^ CD154^+^ T cells, which are unable to secrete IL-2 and IFN-γ following stimulation with S- protein, and weaker humoral immune responses have been reported in asymptomatic individuals with SARS-CoV-2 infection. In the symptomatic group, the higher frequency of SARS-CoV-2-specific CD4^+^ CD154^+^ T cells was significantly correlated with higher serum levels of SARS-CoV-2-specific IgA, IgG and IgM antibodies (141). Different subpopulations of CD4^+^ T cells, such as Th1, Th2, Th17 and regulatory T cells (Treg), have been found to accumulate in COVID-19 patients. In the following, the subpopulations of CD4^+^T cells, as well as CD8^+^ T cells and memory T cell subsets in COVID-19 inflammatory conditions, will be discussed.

### 3.2 Th1 cells

**Figure 2:**
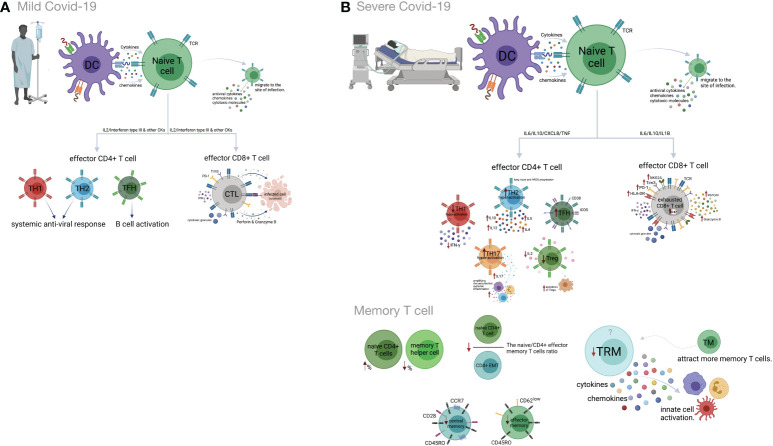
Cell-mediated immune responses during COVID-19 progression in mild versus severe disease. **(A)** Dendritic cells (DCs) promote activation of T cell responses by presentation of SARS-CoV-2 antigenic peptides in major histocompatibility complex (MHC) molecules along with costimulatory interactions and the secretion of chemokines and cytokines. T cell responses can mainly be polarized into T helper (Th) cells and effector cytotoxic cells (CTLs). In mild COVID-19, a higher proportion of Th1 cells plays an important role in defense against the virus by producing interleukin-2 (IL-2) and interferon-gamma (IFN-γ). Th1 cells and the cytokines they secrete activate macrophages, CTLs and natural killer (NK) cells. The Th2 cells stimulate the humoral response and activate eosinophils, basophils and mast cells through IL-4 and IL-5 secretion. The T follicular helper (Tfh) cells are essential for the formation of germinal centers (GCs), B cell maturation and immunoglobulin (Ig) production. CD8^+^ T cells, which coordinate the protective antiviral activities in COVID-19, secrete cytotoxic granules containing perforin and granzyme B as well as IFN-γ in patients who developed mild disease. **(B)** Lymphopenia is a hallmark feature of patients with severe COVID-19. This may be the result of the migration of enormous numbers of T cells to the lungs and other sites of inflammation. Both CD4^+^ and CD8^+^ T cells express strongly CD69, CD38, CD45RO, CD44, HLA-DR, programmed cell death protein-1 (PD-1), T cell immunoglobulin domain and mucin domain-3 (TIM-3) and killer cell lectin-like receptor subfamily C member 1 (NKG2A), which can represent hyperactivated, exhausted or a hypoactivated state of T cells in severe patients. Furthermore, the numbers and functions of regulatory T cells (Treg) are significantly reduced in these patients. Smaller proportions of CD4^+^ and/or CD8^+^ effector cells (CD45RO^+^ CD45RA^−^ CCR7^−^CD28^−^ CD62L^low^), central memory cells (CD45RO^+^ CD45RA^−^ CCR7^+^ CD28^+^) and tissue-resident memory (CD69^+^ CD103^+^ CD49a^+^) T cells were demonstrated in severe COVID-19 patients. Figure was created by BioRender (Toronto, ON, Canada).

Previous studies have indicated that Th1 responses could play protective roles in the early phase of SARS-CoV-2 infection (142). Indeed, highly functional and terminally differentiated effector Th1 cells that eliminate infected target cells have been identified in the early phase of COVID-19 (143, 144). A decreased expression of IFN-γ on CD4^+^ T cells has also been shown in severe COVID-19 cases (145). Moreover, Th1 hypo-activation is associated with a poor prognosis of the disease, and a lower percentage of Th1 cells is observed in severe COVID-19 patients compared to mild patients (146, 147). Earlier investigations suggested that in comparison to mild COVID-19, increased expansion of peripheral neutrophils in severe COVID-19 potentially suppresses Th1 cell differentiation and triggers Th17 cell polarization (148) (**Figure 2**).

### 3.3 Th2 cells

Th2 cells usually mount an appropriate immune response against extracellular pathogens. A recent study showed that Th2 hyper-activation and higher plasma levels of Th2-produced cytokines, including IL-4, -5, -10, and -13, significantly correlated with COVID-19 severity and its related mortality (146, 147). Some studies have highlighted the possible role of Th2 responses in immune-driven lung injury and ARDS progression (142, 143). The association between an increased percentage of Th2 cells and critical care, as well as the poor prognosis, is reported in COVID-19 patients (149) (**Figure 2**).

Th17 cells secrete IL-17 and induce effector cells and inflammatory cytokine production that amplifies uncontrolled systemic inflammation and triggers tissue injury as well as multi-organ failure and death. Several studies have demonstrated that increased frequency and hyper-activation of Th17 cells and subsequent production of pro-inflammatory cytokines are related to the poor outcomes of COVID-19 (35, 142, 145, 148, 150–156). Recently, a higher percentage of GM-CSF^+^ IL-6^+^ CCR6^+^ Th17 cells has been detected in the blood circulation of COVID-19 patients (43). According to new research, Th17 hyperactivation and signaling are significantly correlated with a critical form of COVID-19 (148, 153).

### 3.4 Treg cells

Current evidence indicates smaller amounts of Treg cells in correlation with disease severity in the peripheral blood of COVID-19 patients (115, 157, 158). The frequency of this subset increases during the recovery phase of the disease (159). The decrease in the frequency of Tregs in the peripheral blood of COVID-19 cases might be related to Treg migration into the lungs to resolve lung injury. Besides, there is some evidence of enhanced apoptosis of Tregs due to reduced IL-2 levels in severe compared to mild cases (139, 154, 160–162). A higher frequency of Treg cells in the early phase and a lower frequency in the late phase of SARS-CoV-2 infection could be associated with poor outcomes of COVID-19 (139, 154, 160–162). Accordingly, a decrease in Treg cells along with an increase in Th17 cells is associated with the deregulated pro-inflammatory cytokine secretion in COVID-19 patients (163) (**Figure 2**).

### 3.5 CD8+ T cells

CD8^+^ T cells exert their antiviral activity by killing or inducing apoptosis of infected cells. This occurs by release of cytotoxic granules (154) and secretion of cytokines (164). Several studies reported lower CD8^+^ T cell counts in patients, even with mild or moderate, but especially with severe or critical COVID-19 (160, 165–168). A decreased frequency of CD8^+^ T cells in non-survivors was reported until death (169). The percentage of HLA-DR^+^ PD-1^+^ Tim-3^+^ NKG2A^+^ activated CD8^+^ T cells has been reported to be higher in patients with severe than mild COVID-19 (154, 170, 171). These findings suggest that hyper-active CD8^+^ T cells may play a protective role in the early phase of COVID-19, but rather a pathogenic role in the late phases of the disease due to reduced cytotoxic function and increased cytokine production (160).

### 3.6 Memory T cells

Memory T cells display rapid responses against subsequent infection with similar or related pathogens (172). SARS-CoV-2-specific memory T cell responses in the early recovery phase have been reported recently (138). Compared to mild disease, an increased percentage of naïve CD4^+^T cells and a decreased percentage of memory T helper cells are observed in severe COVID-19 (173). Also, reduced frequencies of CD4^+^ and/or CD8^+^ effector and central memory T cells have been observed in severe compared to mild COVID-19. The naive/effector CD4^+^ memory T cell ratio, an indicator of the differentiation from naive to memory T cells, is reduced in severe cases due to severe impairment of adaptive immunity (14, 174). Tissue-resident memory T cells secrete cytokines and chemokines, resulting in the activation of innate cells and an increase in the recruitment of memory T cells from the periphery (175, 176). Although airway-resident memory T cells mediate protective immune responses against emerging respiratory coronaviruses, the role of this memory subset in the immunopathogenesis of SARS-Cov2 infection should be analyzed further (177). Accordingly, studies on COVID-19 patients, who required ICU treatment, have revealed a lower frequency of tissue-resident memory T cells than effector-memory T cells (178). Likewise, tissue-resident memory-like Th17 cells (TRM17 cells), characterized by an aberrant cytokine profile, are found in the lungs of recovered cases (179). Despite these findings, some studies state that the enrichment of virus-specific CD8^+^ TRM cells in the patients´ respiratory system is positively correlated with the degree of lung damage and even contributes to damage after the recovery (180) (**Figure 2**).

### 3.7 Humoral immunity

In addition to antibody production, B cells display antiviral immune responses through the secretion of inflammatory mediators and T cell activation through antigen presentation (181–183). These cells are a pivotal source of neutralizing antibodies (Abs) that interfere with viral infections, including SARS, MERS, HIV and Ebola. They bind to the virions and prevent their entry into the host cells. Antibodies may activate the complement system and kill infected cells through antibody-dependent cellular cytotoxicity (ADCC) (126, 184, 185). On the other hand, antibodies could also facilitate viral infections by increasing virion uptake *via* Fc or complement receptors. The former is referred to as antibody-dependent enhancement (ADE). ADE has been observed in dengue virus, HIV, influenza, RSV and Ebola, as well as SARS-CoV-2 infections (181, 186–188).

Impaired cellular and humoral immune responses have been reported in COVID-19 patients (5, 189). B cell immune responses to SARS-CoV-2 infection have been found to be stronger in severe than in mild COVID-19. Accordingly, increased clonal expansion of B cells, and frequencies of plasmablasts, complement activation and phagocytosis have been reported in severe cases (190, 191). Similarly to chronic hepatitis B infection, the overall amounts of B cells are large in severe COVID-19 cases. However, the B cell responses are deficient due to decreased clonal diversity. In addition to decreased T cell activity, this is another cause for improper immune responses against infectious agents in elderly individuals (192, 193). Conversely, several studies have reported decreased absolute numbers and frequencies of B cells, as well as lower expression of genes associated with BCR activation signaling, in severe vs. mild COVID-19. This might be due to the consumption of B cells by SARS-CoV-2 in order to inhibit immune activity (2, 83, 154, 194–198).

Also memory B cells are produced after infection with SARS-CoV-2. They include classical CD24^+^ class-switched memory B cells, activated CD24^-^ and natural unswitched CD27^+^ IgD^+^ IgM^+^ subsets. Recently, reduced frequencies of switched and unswitched memory B cells have been observed in severe COVID-19 patients (83, 190, 197, 199–201). In contrast, some studies have reported higher percentages of SARS-CoV2-S-specific memory B cells in severely ill compared to mildly ill patients. The memory B cell levels remain stable several months after disease resolution and provide protection against future exposure (202–206). In one study, a higher proportion of double-negative B cells (IgD^-^ CD27^-^) was reported in severe COVID-19 (199, 207, 208). These cells exacerbate disease progression, possibly by cytokine secretion (209–212). Increased frequencies of CD21^+^and CD21^low/-^ transitional B cells have also been observed in mild to moderate and severe forms of COVID-19 (190, 199).

**Figure 3:**
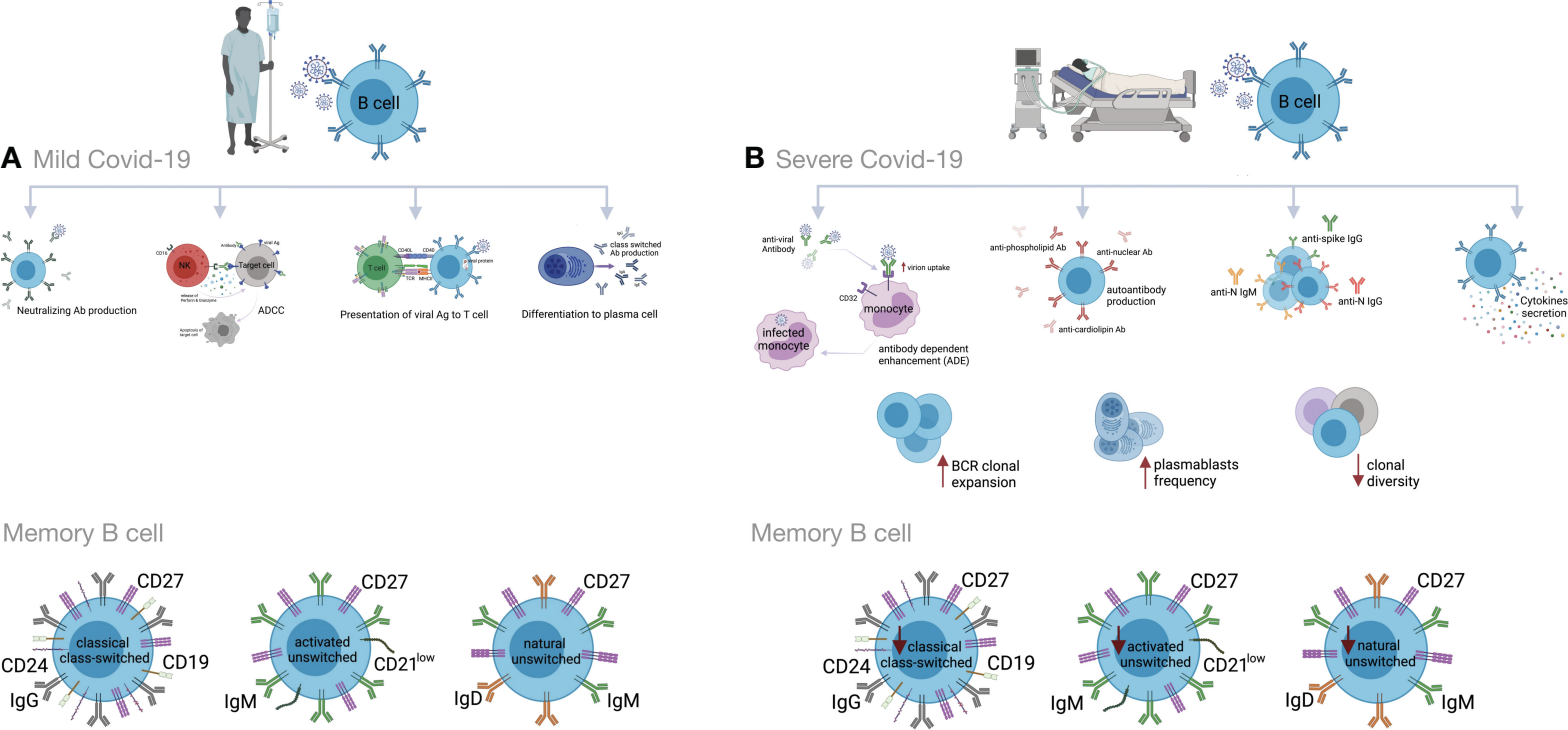
Humoral immune responses during COVID-19 progression in mild versus severe disease. **(A)** During mild COVID-19, B cells contribute to antiviral immune responses in many ways. The first is through production of neutralizing antibodies (Abs), which will bind to the virion and block its entry to the host cells, thus impeding further infection. The second is through a process called antibody-dependent cellular cytotoxicity (ADCC), in which Fc receptor-bearing effector cells can recognize and kill antibody-coated target cells expressing pathogen-derived antigens. The third is through antigen presentation to T cells and activation of cell-mediated immunity. B cells also produce some cytokines to regulate inflammation and activation of adaptive responses. Lastly, B cells can differentiate into antibody-secreting plasma cells, which produce high affinity and class-switched Abs. **(B)** B cell immune responses in severe COVID-19 may exacerbate disease progression by excessive secretion of cytokines and production of autoantibodies. As an adverse effect anti-viral antibodies can facilitate the entry of virus into monocytes/macrophages and granulocytic cells through interaction with Fc and/or complement receptors in a process that is called antibody dependent enhancement (ADE). In addition, increased clonal expansion of B cells, higher frequency of plasmablasts as well as reduced frequency of CD24^+^ switched and CD24^-^CD27^+^ IgD^+^ IgM^+^ unswitched memory B cells have been reported in severe COVID-19 cases. Figure was created by BioRender (Toronto, ON, Canada).

Many studies have shown high titers of antibodies in severe COVID-19 that do not correlate to disease resolution (83, 154, 178, 191, 213–216). Indeed, it has been shown that anti-spike IgG could promote pro-inflammatory responses in M1 macrophages, disruption of endothelial barriers, and microvascular thrombosis (217). This type of thrombotic microangiopathy could be mediated by complement activation. Moreover, anti-SARS-CoV-2 antibodies could enhance virus entry into immune cells through the ADE mechanism (166, 218, 219). In severely ill patients, increased production of autoantibodies, such as antinuclear (ANAs), anti-phospholipid and anti-cardiolipin antibodies, have been associated with poor outcomes (219, 220). They could be due to polyclonal B cell activation or be induced by tissue damage. Also, several studies have reported higher titers of anti-S1- and anti-N- specific IgG and IgM antibodies, as well as neutralizing antibodies (nAbs) in severe vs. mild COVID-19 (199, 221–223). High titers of anti-N Abs have been found to be related to increased virus replication and more severe disease (221) (**Figure 3**).

## 4 Cytokines, interferons and the progression of COVID-19

Cytokines play a major role in many physiological and pathological processes. In the inflammatory context, different immune and non-immune cells, such as monocytes, macrophages, T cells and platelets, as well as endothelial cells, epithelial cells and fibroblasts, produce various cytokines. A cytokine storm, CRS, is defined as an overproduction of cytokines due to an uncontrolled host inflammatory response to different triggers. The triggers could include infections, tissue damage, malignancy, rheumatic disorders and certain drugs. Several studies on severely ill COVID-19 patients have identified significant variations in the serum or plasma levels of cytokines associated with COVID-19 pathogenicity (224, 225). It has been demonstrated that cytokine storm plays an important role in the severity of SARS-CoV-2 infection and lung damage, possibly resulting in ARDS and patient referral to ICU (226, 227). Overproduction of cytokines, including IL-17, IL-7, IL-1β, IL-9, IL-2, IL-10, TNF-α, GM-CSF, G-CSF, IFN-γ, MCP1, MIP1A, MIP1B, CXCL10 and CXCL8, by monocytes and macrophages has been reported in severe COVID-19 cases (182, 228, 229). Moreover, in a study conducted in a group of 52 patients with severe conditions, high plasma concentrations of GM-CSF were reported in COVID-19 patients who required ICU, suggesting a role for GM-CSF in disease severity (182). A study conducted in Taiwan in 2021 compared 40 cytokines in COVID-19 patients with mild, moderate, severe or critical diseases. It was found that the levels of 22 cytokines gradually increased in critical and severe cases compared to moderate ones (230). Evidence indicates that macrophages are the main promoters of cytokine storm during SARS-CoV-2 infection, leading to deleterious clinical manifestations and acute mortality in critically ill patients with COVID-19 (37, 231).

As a broad class of cytokines, interferons (IFNs) are a group of related proteins produced by a variety of cells against viral infection (232). IFNs are classified into three types: type І, type П, and type Ш. Plasmacytoid dendritic cells (pDC) are the main producers of type І IFNs. By binding to their receptors, the interferons induce the expression of IFN-stimulated genes (ISGs), thereby generating an antiviral state by inhibiting virus replication in cells under infection threat (233–235). Several studies have reported that both IFN-β1b and IFN-β1a exert an inhibitory effect on SARS-CoV-2 (236–239). In this regard, an impaired type I IFN response is reported in patients with severely and critically ill COVID-19 compared to those with mild infections. Indeed, patients with lower plasma levels of type I IFN have higher levels of TNF- α and IL-6, increased viral load, and extensive NFκB-driven inflammatory responses (240). A recent study showed that SARS-CoV-2 mRNA-encoded proteins, ORF3b and ORF6, inhibit IFN production and signaling (241). Another study found that high IFN-λ levels during the early stages of SARS-CoV-2 infection were associated with a lower viral load in bronchial aspirates and a better outcome in severely ill COVID-19 patients. In late stages in critically ill COVID-19 patients, however, the serum levels of IFN-α and IFN-λ were found to be decreased compared to those with mild disease (242).

## 5 Therapeutic approaches to COVID-19

Considering that SARS-CoV-2 infection and imbalanced immune responses are the two main drivers of COVID-19 pathogenesis, glucocorticoids and antiviral drugs have been the most commonly used treatments to treat COVID-19 and its complications (243, 244). Safe strategies to limit morbidity and mortality and to improve the efficacy of treatments by finding new therapeutic targets are still urgently required. Current studies have focused on specific means to interfere with SARS-CoV-2 infection (245, 246). Nowadays, several approaches are followed to explore candidates to decrease or subvert inflammatory responses against primary SARS-CoV-2 and mutant strains. Below, the most promising potential targets are reviewed, and an update on the progress of treatments for COVID-19 is provided (**Table 1**).

**Table 1:** Therapeutic approaches in COVID-19.

Therapeutic approaches	Mechanism of action	References
Target for anti-cytokine therapy		
**IL-7**	Immune reconstitution Increased lymphocyte counts	(247, 248)
**IL-6**	Reduced oxygen requirement Reduced CRP level Reduced risk of mortality in ICU Improved lymphocyte production and activity Improved clinical outcomes	(217, 249)
**IL-1**	Reduced oxygen requirement and reduced CRP level Improved clinical condition Reduced white blood cell counts Reduced ferritin, creatinine, procalcitonin and bilirubin levels Increased the PaO2/FiO2 ratio	(250, 251)
**TNF-α**	Reduced inflammatory cytokine production Reduced hyperinflammation	(252, 253)
Interferon therapy		
	Reduced inflammatory cytokine production Improved clinical outcomes	(254, 255)
Cell therapy		
	Reduced inflammatory cytokine production Improved clinical outcomes	(256)
Intravenous immunoglobulin therapy		
	Reduced mortality Improved clinical outcomes	(257)

**Table 2:** Key features of immune responses in mildly versus critically ill COVID-19 patients.

Immune Response Players	Key Features	References
Innate Immune Response Players		
Cytokines	Overproduction of cytokines in severe compared to mild patients can result in detrimental manifestations and mortality	(22, 122)
Interferons	Impaired type I IFN response in patients with severely ill COVID-19 compared to patients with a mild infection	(32)
Complement system	More intense complement activation and inflammatory responses in severely compared to mildly ill patients	(38, 39)
Neutrophils	Higher number of neutrophils, excessive NET formation and ROS production in severely compared to mildly ill patients	(44, 48, 56)
Monocytes	Increased percentage of inflammatory monocytes in severe COVID-19 compared with mild disease	(63)
Macrophages	Macrophages are considered as promoters of cytokine storm and are associated with a high risk of death in severe COVID-19 patients	(73, 74)
Dendritic cells	Decreased number and further impairment in maturation and function of DC in severe compared with mild disease	(83, 84)
ILCs	Decreased frequency of ILC2 in severe COVID-19 in comparison to moderate disease	(89)
Natural killer cells	Decreased number and cytotoxicity of NK cells in severely ill COVID-19 patients	(97, 100)
Basophils	Decreased number and increased expression of PD-L1 on basophils in severe vs. mild COVID-19	(104)
Eosinophils	Decreased number of eosinophils in severe vs. mild disease	(106)
MDSCs	Higher frequency of MDSCs in severely ill COVID-19 patients correlated with decreased specific T cell responses and poor disease outcomes	(107)
NKT cells	Reduced frequency of NKT cells as well as of IFN-γ production in severely ill COVID-19 patients	(129)
Adaptive Immune Response Players		
Th1 cells	Lower percentage of Th1 cells and decreased production of IFN-γ in severe COVID-19	(147, 148)
Th2 cells	Th2 hyper-activation and higher plasma levels of Th2-produced cytokines correlated with disease severity and mortality	(148)
Th17 cells	Increased frequency and hyper-activation of Th17 cells and subsequent production of pro-inflammatory cytokines in critical forms of COVID-19	(149, 151)
Treg cells	Decreased frequency and enhanced apoptosis of Tregs in severe cases compared to mild ones	(156, 158)
CD8+ T cells	Decreased frequency and cytotoxic function of CD8+ T cells in critically ill patients	(166, 167)
Memory T cells	Decreased percentage of memory T helper cells in severe COVID-19	(170)
B cells	Decreased number of B cells and deficient humoral immune responses in severe cases	(185, 186)
Antibodies	Higher titers of antibodies in severe vs. mild patients associated with increased virus replication and a more severe disease	(209)

### 5.1 Anti-cytokine therapy

One approach to treat cytokine storm in COVID-19 is to use traditional anti-inflammatory drugs such as corticosteroids, chloroquine and colchicine (258, 259). However, except for corticosteroids, their use has remained limited. Recently, recombinant cytokines and antibodies or inhibitor molecules against different cytokines and their signaling pathways have been tried or are in the pipeline for production. Below, some of the approaches that are currently or could potentially be applied to manage more serious forms of COVID-19 are described. For the less serious, flu-like illnesses, rest and symptomatic treatment are usually sufficient.

Recombinant human IL-7 (rhIL-7). According to a recent study, recombinant human IL-7 (rhIL-7) administration significantly rescued the immune function in a 74-year-old ICU patient with severe COVID-19 (260). In another study, higher lymphocyte counts without widespread inflammation or pulmonary injury were observed in COVID-19 cases who received IL-7 (247). It has been found that administration of dexamethasone with IL-7 leads to IL7 receptor (IL-7Rα) upregulation and increased IL-7 activity in the more severe stage of COVID-19 (261–263). Another clinical trial (NCT04379076) is currently investigating the effect of CYT107, a commercial derivative of rhIL-7, on the clinical picture and immune reconstitution in severe COVID-19 patients. Accordingly, these results suggest that appropriate administration of IL-7 with or without other agents could be applied to critically ill COVID-19 patients with severe lymphopenia. Because of its lymphopoiesis- stimulating effect, IL-7 has also been suggested as a potential vaccine adjuvant.

### 5.2 Targeting IL-6 signaling

A high concentration of the acute phase response stimulating cytokine IL-6 is related to an increased risk for severe COVID-19. Thus, targeting IL-6 signaling may provide a therapeutic approach for the prevention of aggravated inflammation in SARS-CoV-2 infection (264–266). A retrospective clinical trial showed that treatment with tocilizumab, a monoclonal antibody against IL-6R, reduced oxygen requirements, serum level of CRP, and hospital stays, as well as improving lymphocyte recovery and clinical outcomes in severe or critical COVID-19 (217). Another cohort study demonstrated a decreased mortality in ICU COVID-19 patients who received tocilizumab immediately after ICU admission as compared to those who did not receive early tocilizumab intervention (249). A recent study showed that the treatment of severe COVID-19 patients with sarilumab, a monoclonal antibody against IL-6R, promoted the recovery of cases with mild lung disease (267). In addition, according to a retrospective clinical trial, sarilumab treatment improved clinical symptoms and reduced serum CRP concentrations in most COVID-19 patients (268). A controlled cohort study (NCT04322188) evaluated the impact of siltuximab, a monoclonal antibody against IL-6, on the mortality rate of 30 COVID-19 patients requiring ventilator support. The results showed that siltuximab treatment, in combination with optimal supportive care, reduced the mortality rate of COVID-19 cases as compared to control patients who received only optimal supportive care (269).

### 5.3 Targeting IL-1 signaling

Given the important role of IL-1β in the cytokine storm, several agents targeting IL-1β signaling, including canakinumab and anakinra, have been introduced to clinical COVID-19 treatment trials (250, 270, 271). A retrospective clinical trial among 22 severe/critical COVID-19 patients indicated that >8 days of treatment with anakinra (IL-1 receptor antagonist) led to a reduced requirement for mechanical ventilation, decreased serum CRP levels, and improved clinical conditions in patients compared to the control group (251). Another cohort study found that anakinra treatment reduced several clinical parameters, including temperature, white blood cell count, and plasma levels of ferritin, creatinine, procalcitonin, and bilirubin in COVID-19 patients (272). According to a retrospective study, subcutaneous administration of canakinumab (anti-IL-1β) decreased hyperinflammation and improved PaO2/FiO2 ratio in COVID-19 patients (250). Another cohort study on non-ICU patients with mild or severe COVID-19 who received subcutaneous canakinumab showed that canakinumab treatment significantly increased the PaO2/FiO2 ratio and reduced inflammation (273). Furthermore, other studies have reported the positive effects of canakinumab during SARS-CoV-2 infection (274–276), and six other clinical trials (NCT04348448, NCT04476706, NCT04362813, NCT04365153, NCT04510493, NCT04278404) have been registered to evaluate their potential therapeutic effects in SARS-CoV-2 infection (252).

### 5.4 Targeting TNF-α

Since TNF-α is an initial driver of NF-κB activation and involved in the expression of several pro-inflammatory and anti-apoptotic genes in SARS-CoV-2 infection, its inhibition can attenuate excessive cytokine release and hyperinflammation in COVID-19 (28, 197, 277, 278). Recently, it has been observed that the temporary use of etanercept, a soluble TNF-α receptor fusion protein, reduces hyperinflammation in severe COVID-19 (28). A case report study demonstrated that subcutaneous etanercept administration in a 60-year-old man induced a rapid recovery from COVID-19 and did not cause any signs of respiratory failure (279). A total of four clinical trials of infliximab (anti-TNF-α) (NCT04425538, NCT04734678, NCT04593940, NCT04344249) and two clinical trials with adalimumab, another anti-TNF-α antibody (ChiCTR2000030089, NCT04705844) are ongoing to evaluate its effects in COVID-19 (252).

Interferon therapy: Some patients with life-threatening COVID-19 have been shown to have IgG autoantibodies against type I interferon. Defects, both genetic and acquired, within the IFN pathways have also been shown to predispose to severe COVID-19. Therefore, IFNs have been introduced into clinical trials in an attempt to decrease COVID-19 morbidity and mortality (280). The results of a clinical trial demonstrated that the early triple combination of IFN-1β + lopinavir/ritonavir + ribavirin could reduce clinical symptoms and hospital stay in patients with mild-to-moderate COVID-19 (238). A previous study in China found that lopinavir/ritonavir in combination with IFN-1α reduced the duration of SARS-CoV-2 shedding (281). Other clinical trials and case reports have shown that IFN-α1b and -α2b positively affect cytokine levels in the blood, virus clearance, and clinical symptoms (254, 255). On the other hand, IFNs can also induce adverse effects such as flu-like symptoms, headaches, gastrointestinal reactions, and rashes. In addition, the sustained presence of IFNs could be involved in maintaining local and systemic inflammation and causing tissue damage. The results described above suggest that interferon therapy with antiviral drugs early in the course of infection could help people with mild COVID-19; however, its practicality still remains to be seen.

### 5.5 Cell therapy

While current treatment options for COVID-19 are mainly nonspecific, e.g., the use of dexamethasone or anti-inflammatory agents with significant side effects, the accessibility of other approaches such as remdesivir and tocilizumab (anti-IL-6 receptor) is limited (282). These therapeutic agents have no effects on regenerating damaged tissue structures or functions. Thus, the relevance of cell therapies in the treatment of COVID-19 has received considerable attention (283). Although, particular cell types used in cell therapy for COVID-19 are mesenchymal stem cells (MSCs), NK cells, and T cells, early apoptotic cells, and other cell types are being investigated (284). MSCs exhibit strong immunomodulatory and pluripotential properties that can suppress CD3, CD8, and CD4 T cells and attenuate cytokine secretion (285, 286). MSCs have been used in several clinical trials, including graft vs. host disease (GvHD) (287), type 2 diabetes (288), autoimmune diseases (289), and spinal cord injury (290). Considering that MSCs do not express ACE2 receptors, MSC therapy can accomplish immunomodulatory effects in SARS-CoV-2 infection (291). Previous clinical trials have shown that the intravenous injection of human umbilical cord-derived MSCs is related to an attenuated cytokine storm and improves outcomes in severely ill COVID-19 patients (256). Treatment of severely ill patients who were refractory to steroids with human umbilical cord MSCs results in the healing of pulmonary lesions (292). It has also been reported that a single- dose injection of MSCs in mild, severe, and critical COVID-19 leads to improved clinical outcomes (256). A recent study indicated that COVID-19 patients were extubated approximately 7 days after injection of adipose-derived MSCs (293). Furthermore, a phase 1/2a randomized trial (NCT04355728) found that administration of umbilical cord derived-MSCs for COVID-19 patients with ARDS improved survival following therapy (294).

### 5.6 Intravenous immunoglobulin therapy

IVIG is a natural immunoglobulin pool prepared from the sera of healthy donors. The major component of IVIG is the serum IgG fraction, mainly IgG1 and IgG2 subclasses (295). It is well known that anti-cytokine autoantibodies, including IL-1, IL-6, and IFN-γ autoantibodies, are present in the IVIG of healthy individuals. These antibodies may be related to the anti-inflammatory effects of IVIG in inflammatory and autoimmune disorders (296–298). Previously, the promising effect of IVIG therapy has been reported in SARS and the 2009 H1N1 influenza pandemic (299, 300). Recently, the possible positive effects of IVIG administration during the early phase of COVID-19 have been reported (301). A meta-analysis in three groups of patients with non-severe, severe, or critical illness, including 825 hospitalized COVID-19 patients, indicated that IVIG might be associated with a reduced mortality rate in critically ill patients (302). Further, a significant correlation between IVIG treatment and increased survival rate and decreased COVID-19 progression has been shown (283). Moreover, the potential benefit of IVIG administration along with antiviral drugs and mechanical ventilation in severe COVID-19 patients has been reported (257). Conversely, IVIG administration, along with hydroxychloroquine and lopinavir/ritonavir, in the treatment of severe COVID-19 cases, has not been supported by recent findings (301).

## 6 Conclusion

At this point in the pandemic, further understanding of the SARS-CoV-2 biology and systemic host immune responses can provide information on the processes and mechanisms involved in immune-mediated viral clearance and define specific targets for the treatment of COVID-19. Recent research on the immune mechanisms in COVID-19 refer to the initiation of infection by SARS-CoV-2 accompanied by cellular immune responses, including specifically poly-functional CD4^+^ and CD8^+^ T cell responses, which may become chronic along with immune inflammation. Recovery from tissue injury, prolonged inflammation and deviations in adaptive immune activity may play a role in the postinfectious complications, termed post-COVID. The consequences of deviated immune processes also depend on genetic make-up and environmental risk factors. Due to the SARS-CoV-2 contagiousness, the increased risk of death and the need for an ICU in severe cases of the disease, there is an urgent need for long-term follow-up of the molecular and cellular mechanisms in virus-host communication at all stages of the disease. This information is needed for delineating the optimal management of infected patients in order to prevent the progression to severe forms of the disease. Currently, ongoing large clinical trials based on antiviral and immune-based treatments may soon provide efficient therapeutic agents for COVID-19 patients. In any case, vaccination still remains the best means for preventing severe illness.

## Author contributions

HN, SM and KK conceived the study and wrote the manuscript. HN contributed to the final revision of the manuscript. AT, ZS, MV, PH, NK and MN participated in preparing the first draft and were involved in the final revision of the manuscript. All authors contributed to the article and approved the submitted version.
